# The SARS-CoV-2 Spike Glycoprotein as a Drug and Vaccine Target: Structural Insights into Its Complexes with ACE2 and Antibodies

**DOI:** 10.3390/cells9112343

**Published:** 2020-10-22

**Authors:** Anastassios C. Papageorgiou, Imran Mohsin

**Affiliations:** Turku Bioscience Centre, University of Turku and Åbo Akademi University, 20520 Turku, Finland; mohsin.imran@utu.fi

**Keywords:** SARS, COVID-19, structure, spike, receptor binding, antibodies, glycoprotein

## Abstract

Severe Acute Respiratory Syndrome Coronavirus 2 (SARS-CoV-2), the causative agent of the Coronavirus disease (COVID-19) pandemic, has so far resulted in more than 1.1 M deaths and 40 M cases worldwide with no confirmed remedy yet available. Since the first outbreak in Wuhan, China in December 2019, researchers across the globe have been in a race to develop therapies and vaccines against the disease. SARS-CoV-2, similar to other previously identified *Coronaviridae* family members, encodes several structural proteins, such as spike, envelope, membrane, and nucleocapsid, that are responsible for host penetration, binding, recycling, and pathogenesis. Structural biology has been a key player in understanding the viral infection mechanism and in developing intervention strategies against the new coronavirus. The spike glycoprotein has drawn considerable attention as a means to block viral entry owing to its interactions with the human angiotensin-converting enzyme 2 (ACE2), which acts as a receptor. Here, we review the current knowledge of SARS-CoV-2 and its interactions with ACE2 and antibodies. Structural information of SARS-CoV-2 spike glycoprotein and its complexes with ACE2 and antibodies can provide key input for the development of therapies and vaccines against the new coronavirus.

## 1. Introduction

The Severe Acute Respiratory Syndrome Coronavirus 2 (SARS-CoV-2) pandemic has so far resulted in more than 40 M confirmed cases and >1.1 M deaths worldwide as of 20th October 2020 (https://covid19.who.int/). The deadly respiratory illness caused by SARS-CoV-2 was named Coronavirus disease 19 (COVID-19) by the World Health Organization (WHO) on 11 February 2020. The virus was first discovered in Wuhan, China, in December 2019 [1], and together with the Severe Acute Respiratory Syndrome Coronavirus (SARS-CoV) and the Middle-East Respiratory Syndrome Coronavirus (MERS-CoV) is one of the three coronaviruses that have crossed the species barrier in the 21st century [2]. SARS-CoV and MERS-CoV were reported in 2002 and 2012 with a fatality rate of 10% and 35%, respectively. These zoonotic viruses spread to other species using bats/palm civets and dromedary camels, respectively [3]. However, no certain animal reservoir has been identified yet for SARS-CoV-2 [4].

Since its first emergence, COVID-19 has spread rapidly in more than 212 countries [5] and was declared a global health emergency by WHO on 11 March 2020. No specific treatment is currently available, and a considerable amount of effort is underway to develop vaccines and drugs against the disease.

Structural biology has played a pivotal role in understanding the infection mechanism of the virus and in developing intervention strategies against it. Structures of almost half of the 26 SARS-CoV-2 proteins have been determined by X-ray crystallography and Cryo-Electron Microscopy (Cryo-EM) techniques, some of them within weeks into the pandemic (https://www.ebi.ac.uk/pdbe/covid-19). More than 800 structures of SARS-CoV-2 proteins have been deposited with the Protein Data Bank so far for immediate use by the scientific community. The proteins with the most structures deposited are the two proteases of the virus (>250 structures) and the spike (S) glycoprotein (>80 structures).

The S glycoprotein is one of the four major structural proteins of the virus. It is a solvent-exposed protein responsible for viral entry by binding to the Angiotensin-Converting Enzyme 2 (ACE2) on the human host cells [6]. It has therefore attracted significant attention as a target for the development of vaccines and drugs against SARS-CoV-2. Disruption of the interactions between the S glycoprotein and ACE2 receptor could potentially lead to new therapeutic strategies. Various vaccine efforts are currently based on either its components or the entire protein in its trimeric form [7]. Details of the S glycoprotein interactions with the ACE2 receptor have been revealed by structural biology techniques (Cryo-EM and X-ray crystallography) and have provided valuable information to guide ongoing therapeutic and vaccination efforts. Here, we review the current knowledge on the binding mechanism and the efforts to develop means towards the disruption of S-ACE2 crucial interactions.

## 2. Genome Analysis and Viral Proteins

The draft genome of SARS-CoV-2 was released on 10 January 2020, followed by additional genomes gathered from different patients. The data were deposited in the Global Initiative on Sharing All Influenza Data (GISAID) database, which is primarily used for sharing data on influenza viruses [8]. The availability of the SARS-CoV-2 data allowed detailed genetic evolutionary analyses that revealed that SARS-CoV-2 is a *Betacoronavirus* that belongs to the *Sarbecovirus* subgenus of the Coronaviridae family and is distinct from SARS-CoV [9,10]. The SARS-CoV-2 genome shares 80% of its identity with SARS-CoV and about 96% of its identity with the genome of the bat coronavirus BatCoV RaTG13 [11].

**Figure 1:**
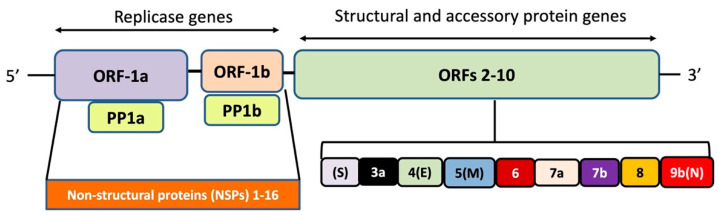
Genome organization of SARS-CoV-2. The polypeptides expressed by open reading frame (ORF)-1a and ORF-1b are cleaved-off to constituent components by the viral proteases papain-like protease (nsp3) and the main protease (nsp5), respectively.

The SARS-CoV-2 genome has 10–12 putative open reading frames (ORFs) that encode non-structural proteins and structural proteins (Figure 1). The non-structural proteins (nsps) are involved in virus processing and replication, while the structural proteins help in the assembly and release of new viral copies. The structural proteins produced are, e.g., spike (S) protein, envelope (E) protein, membrane (M) protein, and nucleocapsid (N) protein [12]. The M protein is the most abundant, while the E protein is the smallest in size amongst all the four structural proteins. More specifically, the M protein acts as a central organizer in assembling and shaping the viral envelope by interacting with other structural proteins. It binds with S and N proteins for the completion of new viral assemblies. The E protein is abundantly expressed in the replication cycle in the infected cells, although a small portion of it is incorporated into the viral envelope and mainly contributes to the viral assembly and budding. The N protein exhibits its functions by interaction with the positive RNA strand of the viral genome, thereby forming a helical ribonucleocapsid complex. It also interacts with other structural membrane proteins during the assembly of virions. The S protein is important for the attachment and entry of SARS-CoV-2 into host cells [13].

**Figure 2:**
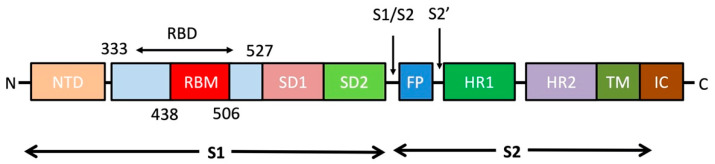
The overall topology of the SARS-CoV-2 S monomer. FP, fusion peptide; HR1, heptad repeat 1; HR2, heptad repeat 2; IC, intracellular domain; NTD, N-terminal domain; SD1, subdomain 1; SD2, subdomain 2; TM, transmembrane region [15].

During the infection, the S protein in its trimeric form is cleaved into subunits S1 (“head”) and S2 (“stalk”) (Figure 2) [14]. S1 is responsible for receptor binding, whilst S2 is responsible for membrane fusion. The ACE2 protein, a key regulator of the renin-angiotensin system [6], acts as the cell entry receptor of SARS-CoV-2 into human cells. S1 protein contains a receptor-binding domain (RBD; ~22 kDa), which interacts with the peptidase domain (PD) of the ACE2 through a receptor-binding motif (RBM) and an N-terminal domain (NTD) whose function is still not well understood. Although the role of NTD is yet unclear, it might be involved in recognition of specific sugar moieties upon initial attachment for the transition of S protein from prefusion to postfusion state as reported in some coronaviruses.

During the binding of S1 to the receptor, S2 undergoes an additional cleavage by host proteases such as Transmembrane Protease Serine 2 (TMPRSS2) through exposure of a second cleavage site (S2′), a process critical for viral infection. This second proteolytic cleavage changes the conformation of the S protein from the prefusion to the postfusion state. Furthermore, a fusion peptide (FP) that penetrates and primes the host cell membrane for fusion is released. Taken together, the coronavirus entry mechanism is a complex process that requires coordinated action between receptor binding and S protein cleavage [16].

The S1/S2 cleavage site has a solvent-exposed loop with multiple arginines (multibasic) that are not found in other SARS-CoV-related coronaviruses [17] apart from human coronavirus OC43, HKU1, and MERS-CoV. It has been suggested that the presence of a multibasic cleavage site may offer advantages in these coronaviruses, and a recombination mechanism has been suggested to explain how the multibasic motif was acquired [18].

A putative furin-recognition motif in SARS-CoV-2, P**RRAR**SV, has been identified as a four amino acid insert (bold letters) close to the S1/S2 cleavage site and at the interface of S1 and S2 [14]. The corresponding sequence in MERS-CoV is P**RSVR**SV, while a similar insert is absent in SARS-CoV [4]. The presence of a furin cleavage site in SARS-CoV-2 has been suggested to facilitate the activation of the S protein for membrane fusion; however, its role may not be as critical as initially thought [19]. An alternative receptor, neuropilin-1 (NRP1), that binds furin-cleaved substrates has been suggested for the virus [20]. A monoclonal antibody against the extracellular b1b2 domain of NRP1 was found to considerably reduce the infectivity of the virus. Due to the involvement of various proteases, it is not surprising that protease inhibitors able to block the proteolytic activation of the S glycoprotein have also been proposed for use as antivirals [21].

Protein glycosylation plays a key role in viral pathogenesis [22]. The S protein, as in other coronavirus S proteins, is highly glycosylated and uses the glycosylation sites to evade the host immune response by shielding specific epitopes from antibody neutralization. The shielding of the receptor binding sites by glycans is a common feature of viral glycoproteins and has been observed in HIV-1 envelope protein (env) and influenza hemagglutinin. There are 22 N-linked glycosylation sites per protomer of the SARS-CoV-2 S protein [23]. Contrary, however, to other S proteins, especially the HIV-1 env where glycosylation accounts for about half of the molecular weight of the protein [24,25], no mannose clusters have been observed in SARS-CoV-1 or SARS-CoV-2 S glycoproteins. Molecular dynamics simulations have suggested that, apart from the shielding, glycans at two sites—N165 and N234—may also offer a conformational stability of the receptor-binding domain towards its recognition by ACE2 [26].

## 3. S protein Structural Details

**Figure 3:**
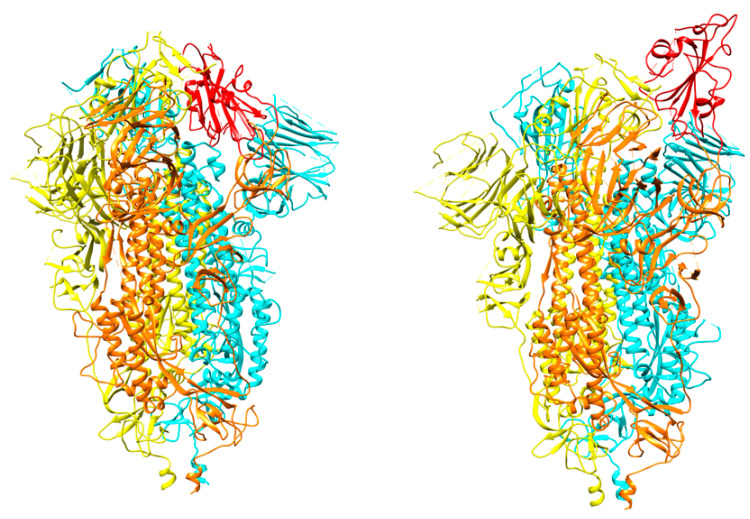
The spike protein trimer in the two conformations: “down” (**left**) and “up” (**right**). The moving receptor-binding domain (RBD) in monomer B is shown in red. Each monomer is colored differently with A, B, and C in yellow, orange, and cyan, respectively (PDB ids 6vxx and 6vyb).

The cryo-EM structure of SARS-CoV-2 S protein was first determined in its prefusion conformation at 3.5-Å resolution [27]. The reported structure revealed an asymmetric trimer and two conformations for one of the RBDs: “up” and “down” (Figure 3). The two conformations are related to the exposure or hiding of key determinants for receptor binding (receptor-accessible and receptor-inaccessible conformations, respectively). ACE2 binding was recently investigated in more detail, resulting in ten structures with the RBDs at different stages of opening [28].

The overall structure shows similarities to that of SARS-CoV S protein (root mean square deviation of 3.8 Å). A key difference was observed in the “down” conformation between the two proteins. In SARS-CoV, the RBD packs tightly against a neighboring monomer. In contrast, the RBD of SARS-CoV-2 is angled towards the cavity of the trimer. Nevertheless, the alignment of individual domains with their counterparts shows high structural homology with only subtle differences. The affinity of SARS-CoV-2 for ACE2 was ~15 nM, ~10 to ~20 times higher than the affinity for SARS-CoV [27]. Other studies, however, have shown that both SARS-CoV-2 and SARS-CoV have similar affinities for ACE2 [29,30].

**Figure 4:**
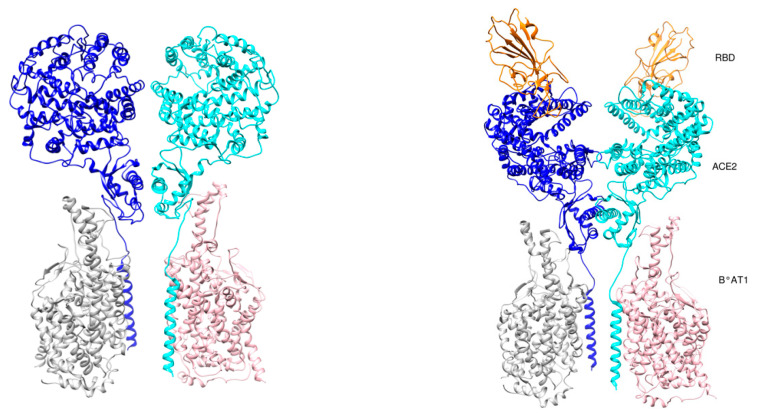
Angiotensin-converting enzyme 2 (ACE2) conformations. (**Left**) Open conformation (PDB id 6m1d). (**Right**) Close conformation (PDB id 6m18). RBD is shown in orange color. ACE2 chains are shown in cyan and blue colors, while B0AT1 chains are shown in pink and grey colors.

In a cryo-EM structure of full-length ACE2 with the amino acid transporter B0AT1 with or without the RBD of SARS-CoV-2, ACE2 was found to form dimers with most of the interactions mediated by its neck domain [31]. In contrast, the peptidase domain (PD) was involved in only a few interactions. Importantly, in one data set the PD was found in a different conformation and the two PDs were separated in the dimers (Figure 4). The two conformations were coined “open” and “closed” [31]. Only the closed conformation of the PD was observed in the RBD-ACE2-B0AT1 ternary complex. Molecular docking of the S trimer to the ACE2 dimer with the bound RBD suggested a plausible simultaneous binding of two S trimers to an ACE2 dimer [31]. 

A pocket that binds linoleic acid (LA), an essential fatty acid, was discovered recently in a cryo-EM structure, raising further possibilities for drug design [32]. The LA binding appears to lock the RBD to the “down” conformation, resulting in a reduced affinity for ACE2. A similar pocket appears also to be present in SARS-CoV and MERS-CoV, based on structure-based sequence alignment [32].

## 4. RBD-ACE2 Interactions

**Figure 5:**
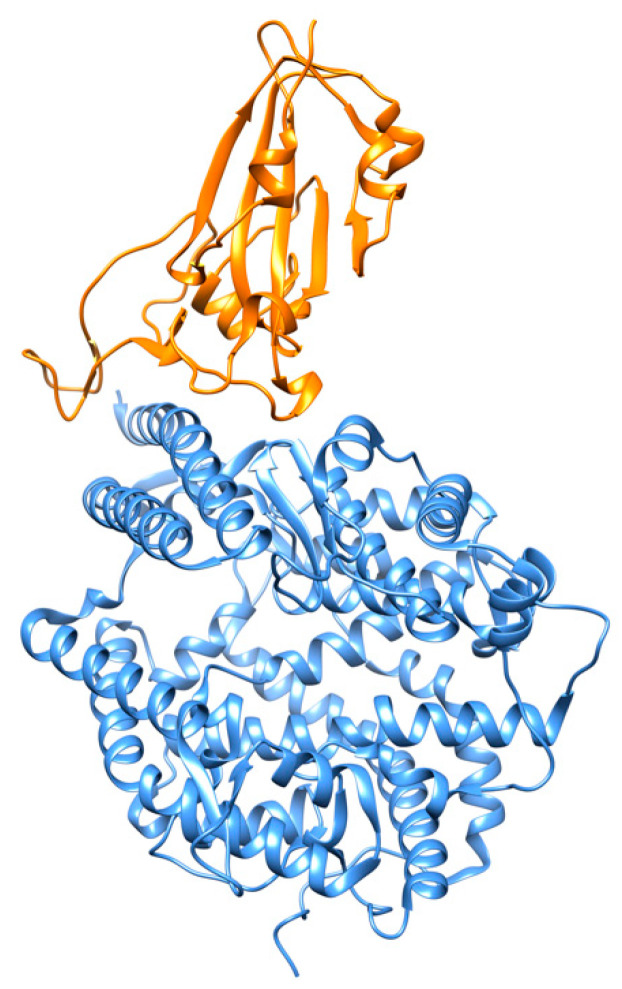
SARS-CoV-2 spike receptor-binding domain (in orange) bound to the ACE2 receptor (in cornflower blue) (PDB id 6m0j) [15].

The interactions of SARS-CoV-2 RBD and ACE2 have been reported at 2.45 Å resolution using X-ray crystallography [15]. The RBD is characterized by a twisted five-stranded antiparallel β-sheet with a long insertion between strands β4 and β7. Most interactions with the N-terminal peptidase domain (PD) of ACE2 are mediated through this long insertion. The N-terminal ACE2 PD has two lobes, forming the peptide substrate binding site between them. The extended RBM in the SARS-CoV-2 RBD contacts the bottom side of the small lobe of ACE2, with a concave outer surface in the RBM that accommodates the N-terminal helix of the ACE2 (Figure 5). The RBD and ACE2 contribute a total of 17 and 20 residues, respectively, at the binding interface. There are 14 residues shared by both SARS-CoV and SARS-CoV-2 RBDs during their binding to ACE2, and of them 8 are identical. Various small molecules, such as hesperidin, a natural compound with anti-inflammatory and anti-oxidant effects isolated from *Citrus aurantium* L., have been proposed as disruptors of the binding interface between the S protein and ACE2 [33]. An overlap of hesperidin with the binding interface of ACE2 was predicted. Modeling efforts based on de novo design have recently led to the synthesis of miniprotein inhibitors that bind to RBD with picomolar affinities and compete with ACE2 [34].

Sequence comparison between the SARS-CoV-2 and SARS-CoV RBDs (~75% sequence identity) with the MERS-CoV RBD (~24% sequence identity) has revealed differences in primary amino acid sequences that lead to different host receptors, i.e., ACE2 for SARS-CoV-2 and SARS-CoV and dipeptidyl peptidase 4 for MERS-CoV [35]. Notably, HCoV-NL63, a common respiratory coronavirus, also uses its RBD to bind ACE2, although there is no structural homology with the RBDs of SARS-CoVs, suggesting a virus-binding hotspot on the shared ACE2 receptor [36]. Other coronaviruses, like HCoV-OC43 and HCoV-HKU1, use their RBD to bind different host receptors, including 9-*O*-acetylated sialic acids [37].

## 5. Examples of Current Targets against SARS-CoV-2

Strategies against coronaviruses include the blocking of the viral entry, inhibition of a virally encoded enzyme, blocking of virus particle formation, or the targeting of a host factor required for replication [38,39]. Although in principle all SARS-CoV-2 proteins could act as potential drug targets, some of them are more likely to be targeted in drug development efforts than others [40]. A large number of small molecules and covalent-bound peptidomimetics that could act as potent inhibitors of the SARS-CoV-2 main protease have been already under intense investigation [41].

Remdesivir targets the viral RNA-dependent RNA polymerase (RdRp), a key enzyme for viral replication, and has been used to assist with COVID-19 treatment in severe cases [42]. Cryo-EM studies of SARS-CoV-2 RdRp in the apo form and in complex with remdesivir and a 50-base template primer RNA showed that remdesivir terminates chain elongation by covalent incorporation into the primer strand at the first replicated base pair [43]. Structural comparisons suggested [43] a highly conserved mode of substrate and inhibitor recognition that could be used for the design of broad-spectrum antiviral drugs based on nucleotide analogs and the improvement of current inhibitors towards higher affinity.

The ribonucleoside analog β-d-N4-hydroxycytidine (NHC; EIDD-1931) has also a broad-spectrum antiviral activity against multiple CoVs as well as increased potency against CoV-bearing resistance mutations to remdesivir [44]. It is believed that NHC acts through the induction of catastrophic mutations. A prodrug of NHC, known as EIDD-2801, has been reported to improve pulmonary function in CoV infections [45].

A protein interaction map between SARS-CoV-2 proteins and human host proteins revealed 332 high-confidence SARS-CoV-2-human protein–protein interactions (PPIs). Among these, 66 druggable human proteins or host factors targeted by 69 compounds (29 FDA-approved drugs, 12 drugs in clinical trials, and 28 preclinical compounds) were identified [46]. Virtual screening of 640 antiviral compounds identified an antiviral polymerase inhibitor, PC786, with good binding affinity against the main protease of the virus and also against the S glycoprotein [47]. A conformational change that may affect the signaling cascade events in the infection process was predicted upon binding of PC786 to the RBD [47].

## 6. Inhibitors against the S Glycoprotein-Mediated Membrane Fusion

**Figure 6:**
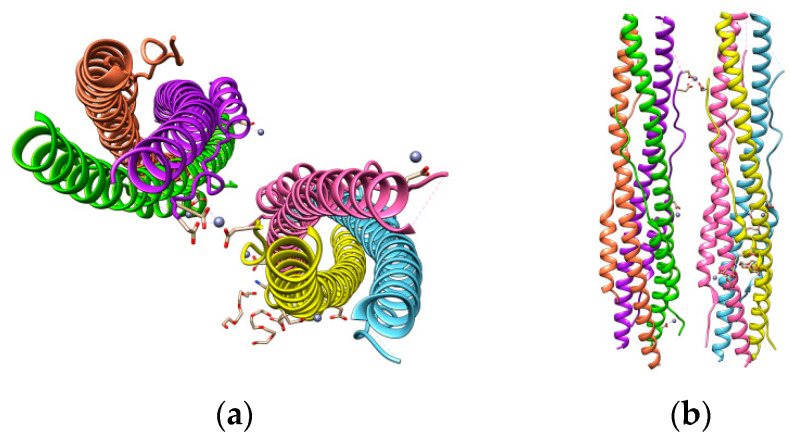
SARS-CoV-2 S protein HR1 and HR2 domains (**a**) and their parallel-antiparallel view (**b**). HR1 helices are colored in yellow, hot pink, and sky blue and those of HR2 in coral, purple, and chartreuse.

After cleavage at the S1/S2 site, the HR1 and HR2 domains of the S2 subunit interact with each other to form a six-helix bundle (6-HB) fusion core. These interactions bring viral and cellular membranes close to each other for fusion and infection [48]. The X-ray crystal structure to 2.90 Å resolution of the 6-HB core of the HR1 and HR2 domains in the SARS-CoV-2 S protein S2 subunit revealed that several mutated amino acid residues in the HR1 domain may be responsible for enhanced interactions with the HR2 domain [49]. The canonical 6-HB structure was found to possess a rod-like shape 115 Å in length and 25 Å in diameter [48]. Three HR1 domains were found entangled with the other three HR2 domains in a parallel-antiparallel manner forming a trimeric coiled-coil center (Figure 6). The interactions between these domains are hydrophobic.

A series of lipopeptides have been developed that could inhibit the fusion process. Amongst them, the lipopeptide EK1C4 was the most potent fusion inhibitor. It was based on the EK1 peptide that was initially developed as a pan-coronavirus fusion inhibitor [48]. Following cholesterol modification, EK1C4 was found to be 226 times more potent against SARS-CoV-2 protein-mediated fusion than its parent EK1 compound. Intranasal application of EK1C4 has shown protections of mice from coronavirus infections and therefore the compound appears promising against the current SARS-CoV-2 as well [49].

## 7. Antibodies Targeting the S Glycoprotein

Neutralizing monoclonal antibodies (MAbs) are potential candidates for use against emerging viruses and for prophylactic and therapeutic treatment against the COVID-19 virus [30,31,32]. Drugs such as ZMapp and MAb114 that bind to Ebola virus S glycoprotein have shown promising therapeutic outcomes by reducing mortality rates to ~50% and ~34% in all patients, respectively [50]. Several vaccine candidates have been developed that target the glycoprotein of the Ebola virus but only one has already completed the phase III of clinical trials [51].

Neutralizing antibodies have already been found in patients of SARS-CoV and MERS-CoV [52,53]. Thus, screening for neutralizing antibodies that target SARS-CoV-2 S glycoprotein has become a priority. Since COVID-19 was declared a pandemic, epitope characterization on the viral RBDs has been particularly important for the development of vaccines, peptide drugs, and inhibitors. The S protein of SARS-CoV-2 shares 76% of its sequence identity with that of SARS-CoV, leading to initial predictions of epitopes. Differences, however, have pointed to a better affinity for SARS-CoV-2. Various structures (cryo-EM and X-ray) are currently available (Table 1).

A description of some of the current structures available is given below:

### 7.1. Antibodies against the RBD

#### 7.1.1. CA1 and CB6

**Figure 7:**
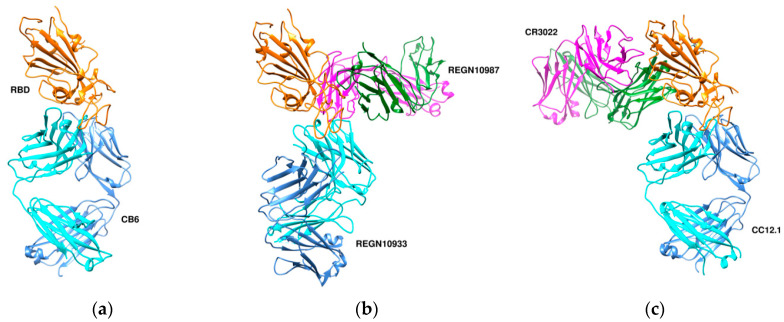
Three different binding modes of neutralizing antibodies. (**a**) Human neutralizing CB6 antibody in complex with SARS-CoV-2 RBD (PDB id 7c01). RBD, H, and L chains are shown in orange, cyan, and cornflower blue colors, respectively. (**b**) REGN antibodies bound to RBD (PDB id 6xdg, (**c**) CR3022 and CC12.1 in complex with ACE2 RBD (PDB id 6xc3). RBD is colored in orange, heavy chains in cyan and green, and L chains in cornflower blue and magenta. The RBD (in orange) is shown in the same orientation in all three complexes.

CA1 and CB6 are able to neutralize SARS-CoV-2 infection in vitro [63]. CB6 was found to have superior neutralization activity to CA1. Besides, CB6 showed inhibition of viral titer and reduction in lung damage when used in rhesus monkeys under prophylactic and treatment settings. The crystal structure of CB6 with the RBD was determined at 2.9 Å resolution (Figure 7a). The entire CB6 light chain as well as most of the heavy chain have structural clashes with the receptor. Binding, however, to the RBD did not induce any structural changes in the RBD.

#### 7.1.2. REGN10933 and REGN10987

Humanized mice and convalescent patients were subjected to generate antibodies against the SARS-CoV-2 spike protein. A large collection of fully-human antibodies was obtained and characterized for binding and neutralization [60]. Two antibodies—REGN10987 and REGN10933—were further selected for structural studies in the presence of the RBD. They were found to bind simultaneously to distinct sites of the RBD with REGN10933 targeting and overlapping with the ACE2 binding site (Figure 7b). On the other hand, REGN10987 was found to bind on the side of the RBD with no overlapping with the ACE2 binding site.

#### 7.1.3. CR3022 and CC12.1

The crystal structure of CR3022, a neutralizing antibody previously isolated from a convalescent SARS patient, in complex with the RBD of the SARS-CoV-2 spike S protein was determined at 3.1-Å resolution [65]. Binding of CR3022 requires that the RBD is in the “up” conformation. CR3022 does not compete with ACE2 for binding to the RBD, therefore binding of CR3022 would not clash with ACE2 binding. Modelling studies, however, using the entire SARS-CoV-2 S protein suggested clashes of the variable and constant region of the antibody with the S2 and N-terminal domain, respectively. Co-crystal structure of the RBD-CR3022 complex with the CC12.1 antibody isolated from a SARS-CoV-2 infected patient revealed the different epitopes between CR3022 and CC12.1 (Figure 7c).

#### 7.1.4. S309

**Figure 8:**
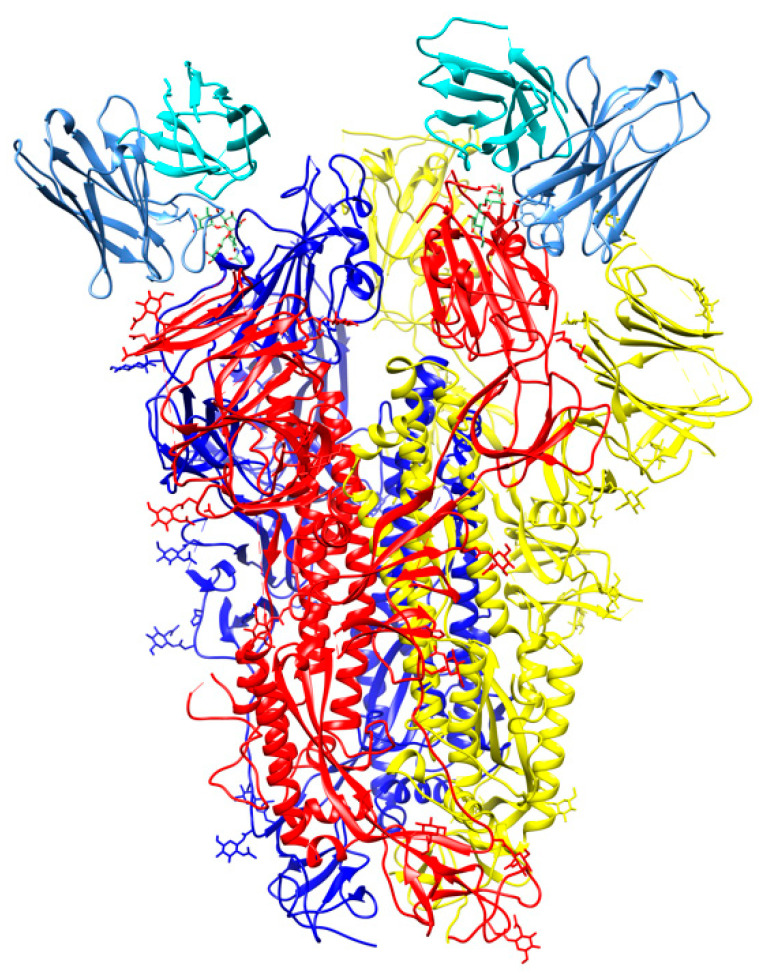
Cryo-Electron Microscopy (Cryo-EM) structure of S309 antibody with the S glycoprotein trimer (PDB id 6wpt). The antibody chains are colored in cyan (L chain) and cornflower blue (H chain). The chains of the S trimer are shown in blue, red, and yellow.

An antibody, named S309, was identified from memory B cells of a SARS-CoV patient in 2003. In neutralization assays, S309 exhibited comparable neutralization potencies against SARS-CoV and SARS-CoV-2. Cryo-EM studies (Figure 8) showed that S309 recognizes a glycan-containing epitope at N343 without competing with receptor binding [57]. It was suggested that neutralization activity may be caused by recruitment of effector mechanisms.

#### 7.1.5. P2B-2F6

**Figure 9:**
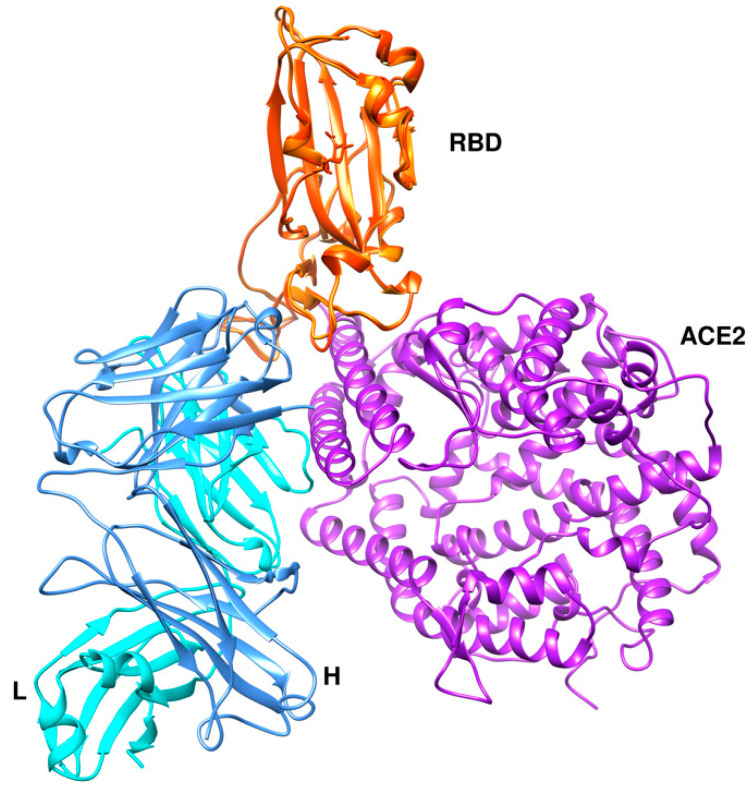
Crystal structure of SARS-CoV-2 P2B-2F6 antibody with RBD (PDB id 7bwj [59] superimposed onto the RBD-ACE2 complex structure (PDB id 6m0j). The RBD is shown in orange color and ACE2 in medium purple. The antibody chains are colored in cyan (L chain) and cornflower blue (H chain).

A set of 206 antibodies was isolated from single B-cells of eight SARS-CoV-2 patients and led to the identification of antibodies that could prevent ACE2 binding to the RBD [59]. Importantly, the antibodies showed no binding to SARS-CoV or MERS-CoV RBDs, although they were able to bind to the trimeric form of their S glycoproteins. The structure of a complex between one of the antibodies, P2B-2F6 Fab, and SARS-CoV-2 RBD revealed interactions of heavy chain residues with the RBD. Structural superposition with the RBD-ACE2 complex showed potential clashes with ACE2, mainly through light chain residues of the Fab (Figure 9). The binding affinity was 5.14 nM, comparable to that of ACE2-RBD binding affinity (4.70 nM). P2B-2F6 Fab could also bind RBD in both “up” and “down” conformations without clashes with the rest of the trimer, as suggested by structural modeling.

#### 7.1.6. B38 and H4

These two antibodies were found to block the binding of the RBD to ACE2 by utilizing different epitopes on the RBD with some partial overlap. The crystal structure of the RBD-B38 complex at 1.9 Å resolution revealed that most residues on the epitope overlap with the RBD-ACE2 binding interface, explaining the blocking effect and neutralizing capacity [66]. There are five complementary determining regions (CDRs): three on the heavy chain and two on the light chain that interact with RBD. The buried surface area between the epitope and the heavy chain is 713.9 Å, and that of light chain is 497.7 Å. Out of 36 RBD residues found to interact with B38, 21 residues interact with the heavy chain and 15 residues interact with the light chain. Sequence alignment has confirmed that 15 out of 36 RBD residues are conserved in the epitope between SARS-CoV-2 and SARS-CoV viruses. The interactions between RBD and B38 were found to be mostly hydrophilic in nature.

### 7.2. Antibodies against the NTD

**Figure 10:**
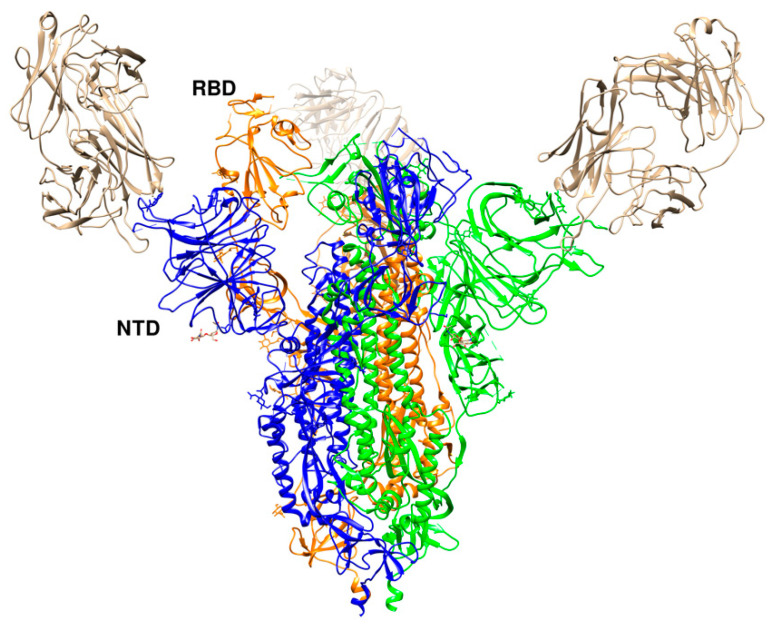
Crystal structure of SARS-CoV-2 RBD with 4A8 (PDB id 7c2l). The RBD in the “up” conformation is labelled. The trimer is colored in blue (B), green (C), and orange (A). The three 4A8 antibodies, each bound to an NTD, are shown in brown.

An antibody, namely 4A8, isolated from convalescent COVID-19 patients showed neutralizing activity against SARS-CoV-2. The epitope of 4A8 was found on the NTD of the S protein as unveiled by the cryo-EM structure of the complex with the S protein to an overall resolution of 3.1 Å (Figure 10) and local resolution of 3.3 Å for the 4A8-NTD interface [54]. No overlap with the RBD was found, leading to speculations that the neutralization potency of 4A8 may stem from a reduction of the conformational flexibility of the S glycoprotein. An antibody, however, against the MERS-CoV found to bind to the NTD of the S protein exhibited some overlap with the binding interface, suggesting that the light chain of the antibody may prevent binding of the DPP4 receptor [67]. The presence of antibodies with neutralizing activities without the need to bind to the RBD could suggest other important mechanisms of SARS-CoV-2 neutralization in addition to the prevention of the virus interactions with the ACE2 receptor.

## 8. Conclusions

The spike glycoprotein has attracted considerable attention owing to its critical role in SARS-CoV-2 cell entry mechanism. Disruption of its interactions with the ACE2 receptor is being pursued as a potential intervention strategy targeting the cell entry of the virus. Neutralizing antibodies have been identified and studied in detail. These neutralizing antibodies have been found to disrupt ACE2 binding to the RBD, while others recognize epitopes away from the RBD-ACE2 binding interface. As a single antibody may not be sufficient and could lead to mutations, the prospect of using a mixture of antibodies has been put forward. A cocktail of neutralizing antibodies that simultaneously bind to the RBD could decrease the chances of the virus to escape even in the event of mutations in response to selective pressure during antibody treatment. Other ways, such as small molecules and miniproteins, that aim to disrupt the function of the S glycoprotein are also under investigation and may lead to the development of novel drugs. There is an increased urgency to develop treatment and long immunity against COVID-19, and the exploitation of the SARS-CoV-2 spike protein as a drug and vaccine target will continue to be an active field in the fight against the COVID-19 pandemic.

## Figures and Tables

**Table 1:** Structures of antibody complexes with S glycoprotein components.

PDB id	Method	Resolution (Å)	Complex Information	Reference
7c2l	Electron microscopy	3.1	4A8 Fab, S1 NTD	[54]
6xcm	Electron microscopy	3.42	S1 RBD, C105 Fab	[55]
6xc7	X-ray diffraction	2.883	CR3022, S1 RBD, CC12.3	[56]
6wpt	Electron microscopy	3.7	S1 RBD, S309 neutralizing antibody	[57]
7byr	Electron microscopy	3.84	Spike glycoprotein trimer, Ab23-Fab	[58]
6xc4	X-ray diffraction	2.341	S1 RBD, CC12.3 antibody	[56]
7c8w	X-ray diffraction	2.77	Sybody MR17, S1 RBD	To be published
7bwj	X-ray diffraction	2.85	S1 RBD, P2B-F26 antibody	[59]
6wps	Electron microscopy	3.1	S309 neutralizing antibody with whole trimer	[57]
6xdg	Electron microscopy	3.9	REGN10987, REGN10933 antibody, S1 RBD	[60]
6xc3	X-ray diffraction	2.698	CR3022, CC12.1, S1 RBD	[56]
7can	X-ray diffraction	2.94	Sybody MR17-K99Y, S1 RBD	To be published
6xc2	X-ray diffraction	3.112	CC12.1 antibody, S1 RBD	[56]
6xey	Electron microscopy	3.25	Ig-like domain-containing protein with the entire S trimer	[61]
7c01	X-ray diffraction	2.88	CB6 antibody, S1 RBD	[62]
6xcn	Electron microscopy	3.66	C105 Fab with the whole S trimer	[55]
6xe1	X-ray diffraction	2.75	CV30 Fab Kappa chain, S1 RBD	[63]
7c8v	X-ray diffraction	2.15	Sybody SR4, S1 RBD	To be published
6w41	X-ray diffraction	3.084	S1 RBD, CR3022 Fab	[64]
7bz5	X-ray diffraction	1.84	B38 neutralizing antibody, S1 RBD	[65]
6zxn	Electron Microscopy	2.9	Alpaca nanobody with S trimer	[66]

## References

[1] Zhu N., Zhang D., Wang W., Li X., Yang B., Song J., Zhao X., Huang B., Shi W., Lu R. (2020). China Novel Coronavirus Investigating and Research Team A Novel Coronavirus from Patients with Pneumonia in China, 2019. N. Engl. J. Med..

[2] Tang D., Comish P., Kang R. (2020). The hallmarks of COVID-19 disease. PLoS Pathog..

[3] Cui J., Li F., Shi Z.-L. (2019). Origin and evolution of pathogenic coronaviruses. Nat. Rev. Microbiol..

[4] Zhang T., Wu Q., Zhang Z. (2020). Probable Pangolin Origin of SARS-CoV-2 Associated with the COVID-19 Outbreak. Curr. Biol..

[5] Bedford J., Enria D., Giesecke J., Heymann D.L., Ihekweazu C., Kobinger G., Lane H.C., Memish Z., Oh M.-D., Sall A.A. (2020). WHO Strategic and Technical Advisory Group for Infectious Hazards COVID-19: Towards controlling of a pandemic. Lancet.

[6] Gheblawi M., Wang K., Viveiros A., Nguyen Q., Zhong J.-C., Turner A.J., Raizada M.K., Grant M.B., Oudit G.Y. (2020). Angiotensin-Converting Enzyme 2: SARS-CoV-2 Receptor and Regulator of the Renin-Angiotensin System: Celebrating the 20th Anniversary of the Discovery of ACE2. Circ. Res..

[7] Baric R.S. (2020). The Current and Future State of Vaccines, Antivirals and Gene Therapies against Emerging Coronaviruses. Front Microbiol..

[8] Zhang Y.-Z., Holmes E.C. (2020). A Genomic Perspective on the Origin and Emergence of SARS-CoV-2. Cell.

[9] Nakagawa S., Miyazawa T. (2020). Genome evolution of SARS-CoV-2 and its virological characteristics. Inflamm. Regen..

[10] Kasibhatla S.M., Kinikar M., Limaye S., Kale M.M., Kulkarni-Kale U. (2020). Understanding evolution of SARS-CoV-2: A perspective from analysis of genetic diversity of RdRp gene. J. Med. Virol..

[11] Zhou P., Yang X.-L., Wang X.-G., Hu B., Zhang L., Zhang W., Si H.-R., Zhu Y., Li B., Huang C.-L. (2020). A pneumonia outbreak associated with a new coronavirus of probable bat origin. Nature.

[12] Pooladanda V., Thatikonda S., Godugu C. (2020). The current understanding and potential therapeutic options to combat COVID-19. Life Sci..

[13] Shang J., Wan Y., Luo C., Ye G., Geng Q., Auerbach A., Li F. (2020). Cell entry mechanisms of SARS-CoV-2. Proc. Natl. Acad. Sci. USA.

[14] Jaimes J.A., Millet J.K., Whittaker G.R. (2020). Proteolytic Cleavage of the SARS-CoV-2 Spike Protein and the Role of the Novel S1/S2 Site. IScience.

[15] Lan J., Ge J., Yu J., Shan S., Zhou H., Fan S., Zhang Q., Shi X., Wang Q., Zhang L. (2020). Structure of the SARS-CoV-2 spike receptor-binding domain bound to the ACE2 receptor. Nature.

[16] Walls A.C., Park Y.-J., Tortorici M.A., Wall A., McGuire A.T., Veesler D. (2020). Structure, Function, and Antigenicity of the SARS-CoV-2 Spike Glycoprotein. Cell.

[17] Hoffmann M., Kleine-Weber H., Pöhlmann S. (2020). A Multibasic Cleavage Site in the Spike Protein of SARS-CoV-2 Is Essential for Infection of Human Lung Cells. Mol. Cell.

[18] Zhou H., Chen X., Hu T., Li J., Song H., Liu Y., Wang P., Liu D., Yang J., Holmes E.C. (2020). A novel bat coronavirus closely related to SARS-CoV-2 contains natural insertions at the S1/S2 cleavage site of the spike protein. Curr. Biol..

[19] Xia S., Lan Q., Su S., Wang X., Xu W., Liu Z., Zhu Y., Wang Q., Lu L., Jiang S. (2020). The role of furin cleavage site in SARS-CoV-2 spike protein-mediated membrane fusion in the presence or absence of trypsin. Signal. Transduct. Target. Ther..

[20] Cantuti-Castelvetri L., Ojha R., Pedro L.D., Djannatian M., Franz J., Kuivanen S., Kallio K., Kaya T., Anastasina M., Smura T. (2020). Neuropilin-1 facilitates SARS-CoV-2 cell entry and provides a possible pathway into the central nervous system. bioRxiv.

[21] Zhou Y., Vedantham P., Lu K., Agudelo J., Carrion R., Nunneley J.W., Barnard D., Pöhlmann S., McKerrow J.H., Renslo A.R. (2015). Protease inhibitors targeting coronavirus and filovirus entry. Antivir. Res..

[22] Raman R., Tharakaraman K., Sasisekharan V., Sasisekharan R. (2016). Glycan–protein interactions in viral pathogenesis. Curr. Opin. Struct. Biol..

[23] Watanabe Y., Allen J.D., Wrapp D., McLellan J.S., Crispin M. (2020). Site-specific glycan analysis of the SARS-CoV-2 spike. Science.

[24] Go E.P., Herschhorn A., Gu C., Castillo-Menendez L., Zhang S., Mao Y., Chen H., Ding H., Wakefield J.K., Hua D. (2015). Comparative Analysis of the Glycosylation Profiles of Membrane-Anchored HIV-1 Envelope Glycoprotein Trimers and Soluble gp140. J. Virol..

[25] Seabright G.E., Cottrell C.A., van Gils M.J., D’addabbo A., Harvey D.J., Behrens A.-J., Allen J.D., Watanabe Y., Scaringi N., Polveroni T.M. (2020). Networks of HIV-1 Envelope Glycans Maintain Antibody Epitopes in the Face of Glycan Additions and Deletions. Structure.

[26] Casalino L., Gaieb Z., Dommer A.C., Harbison A.M., Fogarty C.A., Barros E.P., Taylor B.C., Fadda E., Amaro R.E. (2020). Shielding and Beyond: The Roles of Glycans in SARS-CoV-2 Spike Protein. ACS Cent. Sci..

[27] Wrapp D., Wang N., Corbett K.S., Goldsmith J.A., Hsieh C.-L., Abiona O., Graham B.S., McLellan J.S. (2020). Cryo-EM structure of the 2019-nCoV spike in the prefusion conformation. Science.

[28] Benton D.J., Wrobel A.G., Xu P., Roustan C., Martin S.R., Rosenthal P.B., Skehel J.J., Gamblin S.J. (2020). Receptor binding and priming of the spike protein of SARS-CoV-2 for membrane fusion. Nature.

[29] Corrêa Giron C., Laaksonen A., Barroso da Silva F.L. (2020). On the interactions of the receptor-binding domain of SARS-CoV-1 and SARS-CoV-2 spike proteins with monoclonal antibodies and the receptor ACE2. Virus Res..

[30] Tian X., Li C., Huang A., Xia S., Lu S., Shi Z., Lu L., Jiang S., Yang Z., Wu Y. (2020). Potent binding of 2019 novel coronavirus spike protein by a SARS coronavirus-specific human monoclonal antibody. Emerg. Microbes Infect..

[31] Yan R., Zhang Y., Li Y., Xia L., Guo Y., Zhou Q. (2020). Structural basis for the recognition of SARS-CoV-2 by full-length human ACE2. Science.

[32] Toelzer C., Gupta K., Yadav S.K.N., Borucu U., Davidson A.D., Kavanagh Williamson M., Shoemark D.K., Garzoni F., Staufer O., Milligan R. (2020). Free fatty acid binding pocket in the locked structure of SARS-CoV-2 spike protein. Science.

[33] Wu C., Liu Y., Yang Y., Zhang P., Zhong W., Wang Y., Wang Q., Xu Y., Li M., Li X. (2020). Analysis of therapeutic targets for SARS-CoV-2 and discovery of potential drugs by computational methods. Acta Pharm. Sin. B.

[34] Cao L., Goreshnik I., Coventry B., Case J.B., Miller L., Kozodoy L., Chen R.E., Carter L., Walls A.C., Park Y.-J. (2020). De novo design of picomolar SARS-CoV-2 miniprotein inhibitors. Science.

[35] Tai W., He L., Zhang X., Pu J., Voronin D., Jiang S., Zhou Y., Du L. (2020). Characterization of the receptor-binding domain (RBD) of 2019 novel coronavirus: Implication for development of RBD protein as a viral attachment inhibitor and vaccine. Cell. Mol. Immunol..

[36] Wu K., Li W., Peng G., Li F. (2009). Crystal structure of NL63 respiratory coronavirus receptor-binding domain complexed with its human receptor. Proc. Natl. Acad. Sci. USA.

[37] Tortorici M.A., Walls A.C., Lang Y., Wang C., Li Z., Koerhuis D., Boons G.-J., Bosch B.-J., Rey F.A., de Groot R.J. (2019). Structural basis for human coronavirus attachment to sialic acid receptors. Nat. Struct. Mol. Biol..

[38] Zumla A., Chan J.F.W., Azhar E.I., Hui D.S.C., Yuen K.-Y. (2016). Coronaviruses—Drug discovery and therapeutic options. Nat. Rev. Drug Discov..

[39] Saxena A. (2020). Drug targets for COVID-19 therapeutics: Ongoing global efforts. J. Biosci..

[40] McKee D.L., Sternberg A., Stange U., Laufer S., Naujokat C. (2020). Candidate drugs against SARS-CoV-2 and COVID-19. Pharmacol. Res..

[41] Ullrich S., Nitsche C. (2020). The SARS-CoV-2 main protease as drug target. Bioorg. Med. Chem. Lett..

[42] Lamb Y.N. (2020). Remdesivir: First Approval. Drugs.

[43] Yin W., Mao C., Luan X., Shen D.-D., Shen Q., Su H., Wang X., Zhou F., Zhao W., Gao M. (2020). Structural basis for inhibition of the RNA-dependent RNA polymerase from SARS-CoV-2 by remdesivir. Science.

[44] Agostini M.L., Pruijssers A.J., Chappell J.D., Gribble J., Lu X., Andres E.L., Bluemling G.R., Lockwood M.A., Sheahan T.P., Sims A.C. (2019). Small-Molecule Antiviral β-d-N4-Hydroxycytidine Inhibits a Proofreading-Intact Coronavirus with a High Genetic Barrier to Resistance. J. Virol..

[45] Sheahan T.P., Sims A.C., Zhou S., Graham R.L., Pruijssers A.J., Agostini M.L., Leist S.R., Schäfer A., Dinnon K.H., Stevens L.J. (2020). An orally bioavailable broad-spectrum antiviral inhibits SARS-CoV-2 in human airway epithelial cell cultures and multiple coronaviruses in mice. Sci. Transl. Med..

[46] Gordon D.E., Jang G.M., Bouhaddou M., Xu J., Obernier K., White K.M., O’Meara M.J., Rezelj V.V., Guo J.Z., Swaney D.L. (2020). A SARS-CoV-2 protein interaction map reveals targets for drug repurposing. Nature.

[47] Panda P.K., Arul M.N., Patel P., Verma S.K., Luo W., Rubahn H.-G., Mishra Y.K., Suar M., Ahuja R. (2020). Structure-based drug designing and immunoinformatics approach for SARS-CoV-2. Sci. Adv..

[48] Xia S., Zhu Y., Liu M., Lan Q., Xu W., Wu Y., Ying T., Liu S., Shi Z., Jiang S. (2020). Fusion mechanism of 2019-nCoV and fusion inhibitors targeting HR1 domain in spike protein. Cell. Mol. Immunol..

[49] Xia S., Liu M., Wang C., Xu W., Lan Q., Feng S., Qi F., Bao L., Du L., Liu S. (2020). Inhibition of SARS-CoV-2 (previously 2019-nCoV) infection by a highly potent pan-coronavirus fusion inhibitor targeting its spike protein that harbors a high capacity to mediate membrane fusion. Cell Res..

[50] Davidson E., Bryan C., Fong R.H., Barnes T., Pfaff J.M., Mabila M., Rucker J.B., Doranz B.J. (2015). Mechanism of Binding to Ebola Virus Glycoprotein by the ZMapp, ZMAb, and MB-003 Cocktail Antibodies. J. Virol..

[51] Marzi A., Mire C.E. (2019). Current Ebola Virus Vaccine Progress. BioDrugs.

[52] Jiang L., Wang N., Zuo T., Shi X., Poon K.-M.V., Wu Y., Gao F., Li D., Wang R., Guo J. (2014). Potent Neutralization of MERS-CoV by Human Neutralizing Monoclonal Antibodies to the Viral Spike Glycoprotein. Sci. Transl. Med..

[53] Jiang S., Hillyer C., Du L. (2020). Neutralizing Antibodies against SARS-CoV-2 and Other Human Coronaviruses. Trends Immunol..

[54] Chi X., Yan R., Zhang J., Zhang G., Zhang Y., Hao M., Zhang Z., Fan P., Dong Y., Yang Y. (2020). A neutralizing human antibody binds to the N-terminal domain of the Spike protein of SARS-CoV-2. Science.

[55] Barnes C.O., Anthony P., West J., Huey-Tubman K.E., Hoffmann M.A.G., Sharaf N.G., Hoffman P.R., Koranda N., Gristick H.B., Gaebler C. (2020). Structures of human antibodies bound to SARS-CoV-2 spike reveal common epitopes and recurrent features of antibodies. Cell.

[56] Yuan M., Liu H., Wu N.C., Lee C.-C.D., Zhu X., Zhao F., Huang D., Yu W., Hua Y., Tien H. (2020). Structural basis of a shared antibody response to SARS-CoV-2. Science.

[57] Pinto D., Park Y.-J., Beltramello M., Walls A.C., Tortorici M.A., Bianchi S., Jaconi S., Culap K., Zatta F., De Marco A. (2020). Structural and functional analysis of a potent sarbecovirus neutralizing antibody. bioRxiv.

[58] Cao Y., Su B., Guo X., Sun W., Deng Y., Bao L., Zhu Q., Zhang X., Zheng Y., Geng C. (2020). Potent neutralizing antibodies against SARS-CoV-2 identified by high-throughput single-cell sequencing of convalescent patients’ B cells. Cell.

[59] Ju B., Zhang Q., Ge J., Wang R., Sun J., Ge X., Yu J., Shan S., Zhou B., Song S. (2020). Human neutralizing antibodies elicited by SARS-CoV-2 infection. Nature.

[60] Hansen J., Baum A., Pascal K.E., Russo V., Giordano S., Wloga E., Fulton B.O., Yan Y., Koon K., Patel K. (2020). Studies in humanized mice and convalescent humans yield a SARS-CoV-2 antibody cocktail. Science.

[61] Liu L., Wang P., Nair M.S., Yu J., Rapp M., Wang Q., Luo Y., Chan J.F.W., Sahi V., Figueroa A. (2020). Potent neutralizing antibodies against multiple epitopes on SARS-CoV-2 spike. Nature.

[62] Shi R., Shan C., Duan X., Chen Z., Liu P., Song J., Song T., Bi X., Han C., Wu L. (2020). A human neutralizing antibody targets the receptor binding site of SARS-CoV-2. Nature.

[63] Hurlburt N.K., Wan Y.-H., Stuart A.B., Feng J., McGuire A.T., Stamatatos L., Pancera M. (2020). Structural basis for potent neutralization of SARS-CoV-2 and role of antibody affinity maturation. bioRxiv.

[64] Yuan M., Wu N.C., Zhu X., Lee C.-C.D., So R.T.Y., Lv H., Mok C.K.P., Wilson I.A. (2020). A highly conserved cryptic epitope in the receptor binding domains of SARS-CoV-2 and SARS-CoV. Science.

[65] Wu Y., Wang F., Shen C., Peng W., Li D., Zhao C., Li Z., Li S., Bi Y., Yang Y. (2020). A noncompeting pair of human neutralizing antibodies block COVID-19 virus binding to its receptor ACE2. Science.

[66] Hanke L., Vidakovics Perez L., Sheward D.J., Das H., Schulte T., Moliner-Morro A., Corcoran M., Achour A., Karlsson Hedestam G.B., Hällberg B.M. (2020). An alpaca nanobody neutralizes SARS-CoV-2 by blocking receptor interaction. Nat. Commun..

[67] Zhou H., Chen Y., Zhang S., Niu P., Qin K., Jia W., Huang B., Zhang S., Lan J., Zhang L. (2019). Structural definition of a neutralization epitope on the N-terminal domain of MERS-CoV spike glycoprotein. Nat. Commun..

